# Angiotensin I-Converting Enzyme Inhibitor Activity of Some Plants Used in Thai Indigenous Medicine

**DOI:** 10.3390/plants15132068

**Published:** 2026-07-03

**Authors:** Prattana Sumridpiem, Henrik Balslev, Pimonrat Tiansawat, Oratai Neamsuvan, Hataichanok Pandith, Aussara Panya, Saruda Thongyim, Angkhana Inta

**Affiliations:** 1 Department of Biology, Faculty of Science, Chiang Mai University, Chiang Mai 50200, Thailand; prattanacmu@gmail.com (P.S.); pimonrat.t@cmu.ac.th (P.T.); hataichanok064@gmail.com (H.P.); sapaya116@gmail.com (A.P.); 2 Department of Biology, Aarhus University, Building 1540, Ny Munkegade 116, DK-8000 Aarhus C, Denmark; henrik.balslev@bio.au.dk; 3 Forest Restoration Research Unit (FORRU), Faculty of Science, Chiang Mai University, Chiang Mai 50200, Thailand; 4 Environmental Science Research Center, Faculty of Science, Chiang Mai University, Chiang Mai 50200, Thailand; 5 Faculty of Traditional Thai Medicine, Prince of Songkla University, Songkhla 90110, Thailand; oratai.n@psu.ac.th; 6 Cell Engineering for Cancer Therapy Research Group, Chiang Mai University, Chiang Mai 50200, Thailand; supawadeethongyim@gmail.com; 7 Office of Research Administration, Chiang Mai University, Chiang Mai 50200, Thailand

**Keywords:** *Blumea balsamifera*, *Clerodendrum chinense*, ethnic groups, hypertension

## Abstract

The inhibition of angiotensin-converting enzyme (ACE) to lower angiotensin is important in the treatment of hypertension (HT). ACE inhibitory activity is rarely documented in Thai traditional and indigenous medicine. Here, we evaluated the angiotensin I–converting enzyme inhibitory (ACEi) activity through bio-screening of selected medicinal plant species traditionally used for HT treatment by ethnic communities in northern Thailand, including *Blumea balsamifera* (L.) DC., *Clerodendrum chinense* (Osbeck) Mabb., *Rotheca serrata* (L.) Steane & Mabb., and *Zingiber purpureum* Roscoe. Using an in vitro assay, ethanolic extracts were evaluated for ACE inhibitory activity. Among the four extracts tested, the ethanolic leaf extract of *Blumea balsamifera* was the most effective by reducing ACE activity by 29, 36, and 64% at concentrations of 0.4, 2.0, and 10.0 mg/mL, respectively. The rhizome extract of *Zingiber purpureum* showed the second highest activity, with inhibition rates of 34%, 39%, and 40% at the corresponding concentrations. Cytotoxicity testing in HEK293T kidney cells was conducted to underscore the detectable toxicity under the tested conditions. Interestingly, intercultural and cross-cultural comparisons revealed a degree of agreement in the use of medicinal plants for hypertension treatment. Plant species traditionally used across multiple cultures tended to show higher levels of ACE inhibitory activity, suggesting their potential as candidates for the development of novel anti-hypertensive agents. To our knowledge, this is the first report describing the ACE inhibitory activity of medicinal plant species used for hypertension treatment by ethnic communities in northern Thailand.

## 1. Introduction

High blood pressure or hypertension (HT) is a critical public health problem, and the main cause of more than 50,000 deaths in Thailand each year [1,2]. Among the estimated 13.2 million hypertensive patients, only one third have their blood pressure under control [1]. Blood pressure in the human body is controlled by two interlacing mechanisms following the baroreflexes and the renin–angiotensin–aldosterone system (RAAS) [3]. Renin is produced from the juxtaglomerular apparatus of the kidneys and stimulates the angiotensinogen to produce the inactive decapeptide angiotensin I. The inactive decapeptide angiotensin I is converted to the powerful vasoconstrictor angiotensin II by angiotensin-converting enzyme (ACE). This system regulates the blood pressure by angiotensin II and causes the blood vessels to narrow, which increases the blood pressure. Therefore, the ACE plays an important role in hypertension regulation because the inhibition of ACE promotes a pathway to reduce high blood pressure levels. The common use of ACE inhibitors in managing hypertension involves drugs such as captopril and enalapril [4,5,6]. However, limitations including the various adverse effects (e.g., cough, angioedema), variable patient response, and long-term treatment costs continue to drive the search for alternative or complementary approaches [7,8]. Natural products, particularly plant-derived compounds, represent a rich and underexplored source of bioactive molecules with potential anti-hypertensive activity [9,10]. Many phytochemicals—including flavonoids, terpenoids, and phenolic acids—have been reported to modulate key pathways involved in vascular tone, oxidative stress, and Renin–Angiotensin–Aldosterone System (RAAS) signaling, including ACE inhibition [9,11,12]. Importantly, medicinal plants offer structural diversity and multi-target mechanisms, which may provide advantages over single-target synthetic drugs in complex diseases such as hypertension [13]. Ethnopharmacological knowledge provides a rational framework for prioritizing candidate species for drug discovery [14]. Medicinal plants traditionally used by ethnic communities are often selected through long-term empirical observation and intergenerational knowledge transfer, increasing the likelihood of therapeutic relevance [14,15]. Northern Thailand is home to diverse ethnic groups with rich traditions of herbal medicine, particularly for the management of hypertension and related cardiovascular conditions [15,16]. Cross-cultural consensus in plant usage further strengthens the probability of pharmacological activity, as species independently utilized by multiple communities may reflect reproducible therapeutic effects [14,16].

However, the reports about ACE inhibitory activity of herbal materials used in Thai traditional medicine for treating hypertension or cardiovascular related diseases were rarely documented by Thailand’s Ministry of Public Health, while in both rural and urban Thailand, most hypertensive patients (87%) use the herbal medicine as a standard treatment [17].

Based on this rationale, the present study focuses on the bio-screening of selected medicinal plants traditionally used by ethnic communities in northern Thailand for hypertension treatment by evaluating their angiotensin-converting enzyme (ACE) inhibitory activity and safety profile. Therefore, this work aims to identify promising candidates for further development as novel anti-hypertensive agents and to provide scientific validation for traditional medicinal knowledge. To identify candidate medicinal plants with ACE inhibitory potential in Thailand using ethnopharmacological evidence, we gathered data on traditional use of ACE inhibitory activity plants in Thailand used for treating hypertension and cardiovascular related diseases from original published references between 2014 and 2023, covering three herbal recipes [18,19], 36 medicinal plants [19,20], and eight edible plants [21]. The plants were often used to treat similar or specific conditions related to cardiovascular diseases and hypertension; they were selected for screening ACE inhibitors (Table 1).

The extracts of edible plants that were widely reported as traditionally used for hypertension control were investigated for the ACE inhibitory activity (Table 1). The methanolic extract of *Apium graveolens* L. and *Solanum torvum* Sw. and ethyl acetate extract of *Anacardium occidentale* L. gave high percentages of ACE inhibitory activity at a concentration of 5.0 mg/mL of 82, 76, and 64%, respectively [21]. The Thai traditional medicine recipe called Kam Lung Rad Cha Si was in popular use for treating cardiovascular related diseases and hypertension [18]. All herbal materials were extracted together with aqueous decoction and 95% ethanol maceration. The recipe extracts contained ACE inhibitors that were better than captopril, and their IC50 values were 0.03657 ± 0.00113, 0.03075 ± 0.00104, and 0.00841 ± 0.00085 mg/mL, respectively [18]. However, this recipe was reported in two formulations as decoction and powdering (Supplementary Materials, Table S1). Other Thai traditional medicine recipes called Chan Ta Ha Rue Thai and Pra Sa Chan Daeng were popular and used for treating circulatory system disorders, blood tonic, and for reducing body temperature. Among all the herbal extracts with detected ACE inhibitions (Table 1), *Bouea macrophylla* Griff. and *Dischidia major* (Vahl) Merr. exhibited more than 80% inhibition [19]. In traditional Thai medicinal practice, the healer believed that the main mostly cold taste of that recipe indirectly helped (decreasing over heat in blood circulation’s temperature) the treatment of cardiovascular related diseases and hypertension management [22]. All recipes reported above had a main taste which was cold [23]. These reported ACE inhibitors from traditional Thai medicine recipes may highlight traditional Thai knowledge about hypertension care based on traditional medicinal practice. Moreover, the medicinal plants of traditional Thai medicine that were widely used for treating cardiovascular health conditions such as *Dischidia major* (Vahl) Merr., *Euphorbia antiquorum* L., *Mimusops elengi* L., *Mammea siamensis* (Miq.) T. Anderson, and *Tacca chantrieri* Andre (Table 1), were reported to contain the ACE inhibitors. The ethanolic extracts of *Mammea siamensis* (Miq.) T. Anderson flowers and *Senna garrettiana* (Craib) H.S.Irwin & Barneby heartwood at the concentration of 1 mg/mL showed high ACE inhibitor activity of more than 90% [20]. However, variation in phytochemical profiles among different plant organs may reflect the complexity of secondary metabolites, including differences in their concentrations and the effects of the extraction solvent [19,20]. Among different parts of the same species, the ethanolic extracts of root and gall from *Dischidia major* at the concentration of 1 mg/mL showed ACE inhibitor activity of 43 and 81% (Table 1), respectively.

Based on the information above, there were few important ACE inhibitory activities reported in Thailand. Additionally, the documentation focused on the ACE inhibitory activity for treating hypertension in Thai indigenous medicine. Medicinal plants that are traditionally used by multiple cultures over long time periods in hypertension treatment are more likely to be pharmacologically active and they would be good candidates in anti-hypertensive drug discovery [16]. Therefore, this research aimed to evaluate the angiotensin I-converting enzyme inhibitor (ACEi) activity by bio-screening and evaluating their safety profile by cytotoxicity analysis of some prominent species used for hypertension treatment among the ethnic communities in northern Thailand. Four species that are widely used to treat hypertension with the high degree of agreement value among ethnic groups in Chiang Mai province [16] were investigated: *Clerodendrum chinense* (Osbeck) Mabb. (Lamiaceae), *Blumea balsamifera* (L.) DC. (Asteraceae), *Zingiber purpureum* Roscoe (Zingiberaceae), and *Rotheca serrata* (L.) Steane & Mabb. (Lamiaceae).

**Table 1:** Medicinal plants used in traditional Thai medicine recipe and Thai edible plants with ACE inhibitory activity reported.

Scientific Name	Family Name	Part Used (Method)	Solvent Used (Type of Extraction)	ACE Inhibitory Activity (%), Concentration (mg/mL)	[Reference] Used Type
1. *Abroma augusta* (L.) L.f.	Malvaceae	Seeds (B)	Ethanol 95% (A)	No inhibitory activity, 1 mg/mL	[20] Traditional Thai medicine
2. *Albizia myriophylla* Benth.	Fabaceae	Stems (B)	Ethanol 95% (A)	No inhibitory activity, 1 mg/mL	[20] Traditional Thai medicine
3. *Anacardium occidentale* L. *	Anacardiaceae	Leaves (A)	Ethyl acetate and methanol	64.2%, 5 mg/mL	[21] Thai edible plants
4. *Angelica dahurica* (Hoffm.) Benth. and Hook.f. ex Franch. & Sav.	Apiaceae	Roots (C)	Ethanol 70%	No inhibitory activity%, 1 mg/mL	[19] Chan Ta Ha Rue Thai recipe
5. *Apium graveolens* L.	Apiaceae	Whole (A)	Ethyl acetate and methanol	82.3%, 5 mg/mL	[21] Thai edible plants
6. *Artemisia annua* L.	Asteraceae	Roots (C)	Ethanol 70%	2.91 ± 0.25%, 1 mg/mL	[19] Chan Ta Ha Rue Thai recipe
7. *Biancaea sappan* (L.) Tod.	Fabaceae	Heartwood (C)	Ethanol 70%	36.99 ± 0.29%, 1 mg/mL	[19] Pra Sa Chan Daeng recipe
8. *Bouea macrophylla* Griff.	Anacardiaceae	Roots (C)	Ethanol 70%	80.86 ± 0.32%, 1 mg/mL	[19] Pra Sa Chan Daeng recipe
10. *Carissa spinarum* L.	Apocynaceae	Heartwood (C)	Ethanol 70%	No inhibitory activity, 1 mg/mL	[19] Chan Ta Ha Rue Thai recipe
11. *Calamus longisetus* Griff.	Arecaceae	Stems (C)	Ethanol 70%	60.50 ± 0.99%, 1 mg/mL	[19] Chan Ta Ha Rue Thai recipe
12. *Citrus aurantifolia* (Christm.) Swingle *	Rutaceae	Roots(C)	Ethanol 70%	17.01 ± 0.57%, 1 mg/mL	[19] Pra Sa Chan Daeng recipe
13. *Cuminum cyminum* L.	Apiaceae	Seeds (B)	Ethanol 95% (A)	43.4 ± 0.66%, 1 mg/mL	[20] Traditional Thai medicine
14. *Dendrobium crumenatum* Sw.	Orchidaceae	Stems (C)	Ethanol 70%	21.32 ± 1.01%, 1 mg/mL	[19] Chan Ta Ha Rue Thai recipe
15. *Dischidia major* (Vahl) Merr.	Apocynaceae	Galls (B)	Ethanol 95% (A)	43.4 ± 0.66%, 1 mg/mL	[20] Traditional Thai medicine
		Roots (C)	Ethanol 70%	81.19 ± 0.17%, 1 mg/mL	[19] Chan Ta Ha Rue Thai recipe
16. *Dracaena cochinchinensis* (Lour.) S.C. Chen	Asparagaceae	Heartwood (C)	Ethanol 70%	57.60 ± 0.81%, 1 mg/mL	[19] Pra Sa Chan Daeng and Chan Ta Ha Rue Thai recipe
17. *Enhalus acoroides* (L.f.) Royle	Hydrocharitaceae	Rhizomes (C)	Ethanol 70%	63.21 ± 0.73%, 1 mg/mL	[19] Chan Ta Ha Rue Thai recipe
18. *Euphorbia antiquorum* L.	Euphorbiaceae	Stems (B)	Ethanol 95% (A)	No inhibitory activity, 1 mg/mL	[20] Traditional Thai medicine
		Heartwood (C)	Ethanol 70%	16.91 ± 0.51%, 1 mg/mL	[19] Chan Ta Ha Rue Thai recipe
19. *Ficus racemosa* L. *	Moraceae	Fruits (A)	Ethyl acetate and methanol	Lower than 60%, 5 mg/mL	[21] Thai edible plants
20. *Foeniculum vulgare* Mill.	Apiaceae	Seeds (B)	Ethanol 95% (A)	No inhibitory activity, 1 mg/mL	[20] Traditional Thai medicine
21. *Gynura pseudochina* (L.) DC.	Asteraceae	Rhizomes (C)	Ethanol 70%	19.95 ± 0.80%, 1 mg/mL	[19] Chan Ta Ha Rue Thai recipe
22. *Glycyrrhiza glabra* L.	Fabaceae	Roots (C)	Ethanol 70%	67.51 ± 0.45%, 1 mg/mL	[19] Chan Ta Ha Rue Thai recipe
23. *Heliciopsis terminalis* (Kurz) Sleumer	Proteaceae	Roots (C)	Ethanol 70%	81.19 ± 0.17%, 1 mg/mL	[19] Chan Ta Ha Rue Thai recipe
24. *Houttuynia cordata* Thumb.	Saururaceae	Aerial (A)	Ethyl acetate and methanol	Lower than 60%, 5 mg/mL	[21] Thai edible plants
25. *Jasminum sambac* (L.) Aiton.	Oleaceae	Flowers (B)	Ethanol 95% (A)	No inhibitory activity, 1 mg/mL	[20] Traditional Thai medicine
		Flowers (C)	Ethanol 70%	No inhibitory activity, 1 mg/mL	[19] Chan Ta Ha Rue Thai and Pra Sa Chan Daeng recipe
26. *Kaempferia galanga* L.	Zingiberaceae	Rhizomes (C)	Ethanol 70%	No inhibitory activity%, 1 mg/mL	[19] Pra Sa Chan Daeng recipe
27. *Conioselinum anthriscoides* (H.Boissieu) Pimenov & Kljuykov *	Apiaceae	Rhizomes (B)	Ethanol 95% (A)	No inhibitory activity, 1 mg/mL	[20] Traditional Thai medicine
		Rhizomes (C)	Ethanol 70%	14.51 ± 0.87%, 1 mg/mL	[19] Pra Sa Chan Daeng recipe
28. *Mammea siamensis* (Miq.) T.Anderson	Calophyllaceae	Flowers (B)	Ethanol 95% (A)	97.50 ± 0.20%, 1 mg/mL	[20] Traditional Thai medicine
		Flowers (C)	Ethanol 70%	70.85 ± 0.15%, 1 mg/mL	[19] Chan Ta Ha Rue Thai and Pra Sa Chan Daeng recipe
29. *Mesua ferrea* L.	Calophyllaceae	Flowers (B)	Ethanol 95% (A)	9.73 ± 0.09%, 1 mg/mL	[20] Traditional Thai medicine
		Flowers (C)	Ethanol 70%	74.98 ± 0.20%, 1 mg/mL	[19] Chan Ta Ha Rue Thai and Pra Sa Chan Daeng recipe
30. *Mimusops elengi* L.	Sapotaceae	Flower (B)	Ethanol 95% (A)	9.5 ± 0.30%, 1 mg/mL	[20] Traditional Thai medicine
		Heartwood/ Rotten wood (C)	Ethanol 70%	79.06 ± 0.55%, 1 mg/mL	[19] Chan Ta Ha Rue Thai recipe
31. *Morinda citrifolia* L.	Rubiaceae	Leaves (A)	Ethyl acetate and methanol	Lower than 60%, 5 mg/mL	[21] Thai edible plants
32. *Moringa oleifera* Lam.	Moringaceae	Leaves (A)	Ethyl acetate and methanol	Lower than 60%, 5 mg/mL	[20] Traditional Thai medicine
33. *Myristica fragrans* Houtt.	Myristicaceae	Heartwood (C)	Ethanol 70%	57.27 ± 0.35%, 1 mg/mL	[19] Chan Ta Ha Rue Thai and Pra Sa Chan Daeng recipe
34. *Nelumbo nucifera* Gaertn.	Nelumbonaceae	Stamens (C)	Ethanol 70%	65.21 ± 0.61%, 1 mg/mL	[19] Chan Ta Ha Rue Thai and Pra Sa Chan Daeng recipe
35. *Picrorhiza kurroa* Royle ex Benth.	Plantaginaceae	Rhizomes (C)	Ethanol 70%	54.24 ± 1.18%, 1 mg/mL	[19] Chan Ta Ha Rue Thai recipe
36. *Senna garrettiana* (Craib) H.S. Irwin & Barneby	Fabaceae	Heartwood (B)	Ethanol 95% (A)	92.20 ± 2.07%, 1 mg/mL	[20] Traditional Thai medicine
37. *Sophora exigua* Craib	Fabaceae	Roots (C)	Ethanol 70%	67.18 ± 0.26%, 1 mg/mL	[19] Chan Ta Ha Rue Thai recipe
38. *Tacca chantrieri* Andre	Dioscoreaceae	Leaves (A)	Ethyl acetate and methanol	Lower than 60%, 5 mg/mL	[21] Thai edible plants
		Whole plant (C)	Ethanol 70%	76.05 ± 0.23%, 1 mg/mL	[19] Chan Ta Ha Rue Thai recipe
39. *Tarenna hoaensis* Pit.	Rubiaceae	Heartwood (C)	Ethanol 70%	No inhibitory activity, 1 mg/mL	[19] Chan Ta Ha Rue Thai recipe
40. *Terminalia bellirica* (Gaertn.) Roxb. *	Combretaceae	Fruits (B)	Ethanol 95% (A)	No inhibitory activity, 1 mg/mL	[20] Traditional Thai medicine
41. *Terminalia chebula* Retz. *	Combretaceae	Fruits (B)	Ethanol 95% (A)	No inhibitory activity, 1 mg/mL	[20] Traditional Thai medicine
42. *Solanum torvum* Sw.	Solanaceae	Fruits (A)	Ethyl acetate and methanol	76.2%, 5 mg/mL	[21] Thai edible plants
43. *Urceola minutiflora* (Pierre) D.J. Middleton	Apocynaceae	Stems (C)	Ethanol 70%	79.46 ± 0.91%, 1 mg/mL	[19] Chan Ta Ha Rue Thai recipe

Scientific name and family name: from original data sources and updated to follow *World Flora Online* (Available online: https://www.worldfloraonline.org/ [accessed on 12 June 2026]), (*) name was changed. Preparation method for plant part used: (A) dried in hot air oven at 50 °C for 24 h; (B) dried plants from the herbal drugstore; and (C) the herbs were ground into a fine powder (50 mesh) and kept at −20 °C until extraction. Type of extraction: (A) ultrasound-assisted extraction.

## 2. Results

### 2.1. Angiotensin I–Converting Enzyme Inhibitory (ACEi) Activity of Herb Extracts

**Figure 1:**
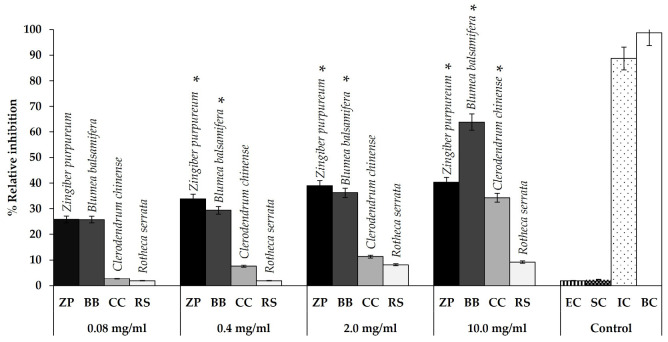
Relative ACE inhibition ratio (%) of ethanolic extracts of plant at different concentrations (value = mean ± SD, *n* = 3). Significant differences were determined by Mann–Whitney U test: *p* value < 0.05 (*). ZP = *Zingiber purpureum*; BB = *Blumea balsamifera*; CC = *Clerodendrum chinense*; RS = *Rotheca serrata*; EC = enzyme control; SC = solvent control; and IC = inhibitor control.

*Zingiber purpureum* (ZP) and *Blumea balsamifera* (BB) produced strong inhibitory effects at all extract concentrations from 2.0 to 10.0 mg/mL (Figure 1). The IC_50_ values were 19.03 and 6.15 mg/mL, respectively. *Clerodendrum chinense* (CC) and *Rotheca serrata* (RS) extracts produced weak inhibitory effects with IC_50_ values of 15.22 and 70.98 mg/mL, respectively. Angiotensin I was converted to Angiotensin II, which produced a relative inhibition of enzyme control (EC) equal to 1.9% as the baseline of hypertension. The bar of inhibitor control (IC), as the positive control, represented the hypertension group that received the anti-hypertensive drug (captopril) (Figure 1). The drug reacted by binding to ACE and then it blocked the conversion of Angiotensin I to Angiotensin II. Thus, the percentage relative inhibition of IC was up to 89% which is taken as the percent standard inhibition for this research. Then, we can observe the results of sample groups (ZP, BB, CC, and RS extract) by comparing them to the EC and IC groups (Figure 1) follows:

*Zingiber purpureum* extracts tended to have moderate inhibitory effect on ACE activity. These values differed significantly from the enzyme control group (*p* value < 0.05).

*Blumea balsamifera* extracts had moderate to good inhibitory effect on ACE activity, especially the leaf extracts, which were significantly different from the enzyme control group (*p* value < 0.05).

*Clerodendrum chinense* leaf extracts had a poor inhibitory effect on ACE activity, only the leaf extracts at concentration had some inhibition which was significantly different from the EC group (*p* value < 0.05).

*Rotheca serrata* leaf extracts had a very poor inhibitory effect on ACE activity because there was no significant difference when compared to the EC group (*p* value < 0.05).

**Figure 2:**
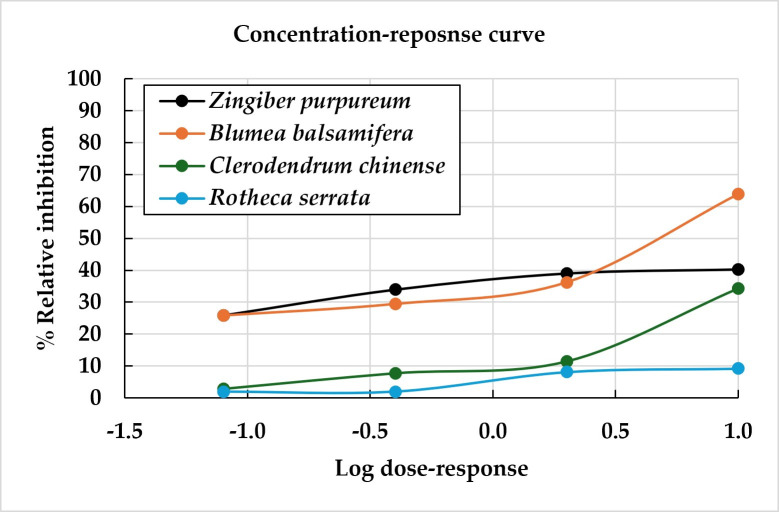
Concentration–response curve.

The concentration-effect curves (Figure 2) to allow for a comparison of the efficacy and potency of the different extracts. Relationship between % relative inhibition and log-dose response demonstrated a positive relationship between extract concentration and ACE inhibitory activity, indicating that increasing concentrations of the extracts result in greater enzyme inhibition.

*Blumea balsamifera* (orange line) showed the highest inhibitory activity, increasing markedly at the highest dose to approximately 64% relative inhibition. *Zingiber purpureum* (black line) exhibited a moderate inhibitory effect, gradually increasing from about 26% to 40% as the dose increased. *Clerodendrum chinense* (green line) demonstrated a dose-dependent increase, with inhibition rising from approximately 2% to 34% at the highest concentration. *Rotheca serrata* (blue line) showed the lowest activity, remaining below 10% relative inhibition across all tested doses.

### 2.2. Cytotoxicity of Herb Extracts in HEK293T Kidney Cells

**Figure 3:**
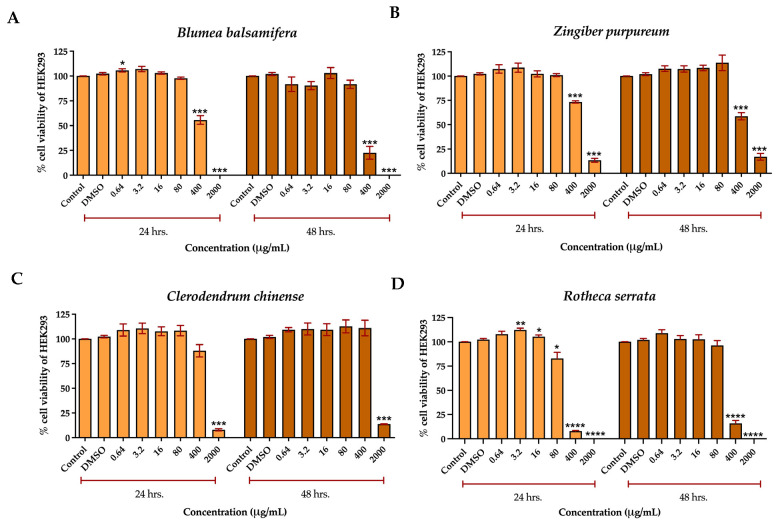
The cytotoxicity of the herb extract in HEK293T kidney cells. Cells were treated with extracts of *Blumea balsamifera* (**A**), *Zingiber purpureum* (**B**), *Clerodendrum chinense* (**C**), and *Rotheca serrata* (**D**). The cells were harvested and their viability was determined at 24 and 48 h after treatment by PrestoBLUE™ cell viability reagent. The results are presented as percentage viability relative to untreated control cells (100%). Asterisks indicate statistical significance as follows: (*) = *p* < 0.05; (**) = *p* < 0.01; (***) = *p* < 0.001 and (****) = *p* < 0.0001.

The cytotoxicity test was performed to support the safety of the herb extract in HEK293T kidney cells. Various concentrations of the extracts were prepared and added to the cells. The cell viability was determined according to the redox capacity of the living cells and calculated as the % cell viability relative to that of non-treatment control. The result showed that the herb extracts caused the cytotoxicity of HEK293T in dose dependent manner. The 24 h treatment of *Clerodendrum chinense*, *Rotheca serrata*, *Zingiber purpureum*, and *Blumea balsamifera* extracts with the concentrations higher than 0.4 mg/mL caused the significant toxicity to the cells by reducing the cell viability (Figure 3).

## 3. Discussion

Extracts from *Blumea balsamifera*, *Zingiber purpureum*, *Clerodendrum chinense*, and *Rotheca serrata* were effective, which is consistent with previous research on ACE inhibitory activity. However, the final concentration of ethanolic plant extract (or dose–response) which had previously been reported on ACE inhibitors in Thailand showed inhibitory activity at a concentration of 1 and 5 mg/mL, respectively (Table 1). The IC_50_ values were not available for most studies [19,20]. Therefore, the final concentration of this study preferred a concentration of 10.0 mg/mL based on a five-fold dilution (Figure 1).

Among the extracts from the four plants that were investigated for their ACE inhibitory activity, the ethanolic extract of *B. balsamifera* gave the highest percentage of ACE inhibitory activity (63.93%, at a concentration of 10 mg/mL) with an IC_50_ value of 6.15 mg/mL, followed by *Z. purpureum* (40.27%, at a concentration of 10 mg/mL) with an IC_50_ value of 19.03 mg/mL. A dose-dependent increase in ACE inhibitory activity was observed for most plant extracts. Among the tested species, *Blumea balsamifera* exhibited the strongest inhibitory effect, reaching approximately 64% relative inhibition at the highest concentration. The other plant extracts showed only weak inhibitory activity with a percentage of ACE inhibitory activity lower than 40%, each one tested at a concentration of 10 mg/mL (Figure 1). However, the potential overlap between ACE inhibitory activity and cytotoxicity should be taken into consideration. The biological activity and reduced cell viability may occur within overlapping concentration ranges. Based on the fact that IC_50_ values of all plant extracts were substantially higher than the concentration that caused significant cytotoxicity (>0.4 mg/mL). This observed in vitro ACE inhibitory activity of the plant extracts may reflect a non-specific effect, with cytotoxicity being detected at active concentrations above 0.4 mg/mL.

**Figure 4:**
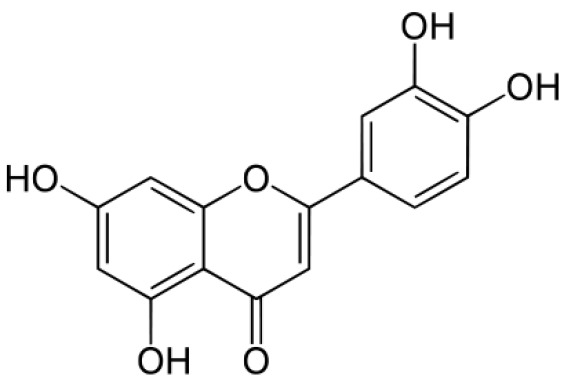
Luteolin [24].

**Figure 5:**
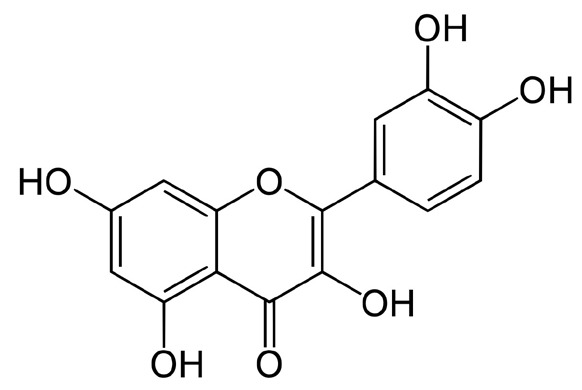
Quercetin [24].

Bioactive compounds from *B. balsamifera* leaves had been reported to produce ACE inhibition in vitro [6]. The aqueous extract or tea preparation of 3 g leaf powder in 180 mL of distilled water at 100 °C for 5 min showed inhibitory activity on rabbit lung ACE [6]. The relative inhibitory activity of the oven-dried tea on ACE was observed as a mean inhibitory activity of 70% while the relative inhibitory activity of the air-dried tea on ACE was observed as a mean inhibitory activity of 211%. The inhibitory activity of the air- dried tea was three times higher compared to that of the oven-dried tea [6]. Bioactive compounds in these plant leaves such as flavonoids, terpenoids [6], quercetin and luteolin [24] had been previously reported (Table 2, Figure 4 and Figure 5). The chemical structure diagrams of Figure 4 and Figure 5 were created by MolView software. Available online: https://molview.org/ (accessed on 12 June 2026). Our evidence provided a valuable foundation for effective screening of the new drugs from traditional medicinal sources. In northern Thailand, *B. balsamifera* leaves were the most commonly used traditional plant drug for treating HT as observed in multiple villages of five different ethnic groups (Hmong, Karen, Lisu, Tai Yai, and Tai Yuan). In the northern Chiangmai province [16] folk healers used *B. balsamifera* leaves for treating hypertension and the same was the case in the southern Songkhla province [25]. The data documented similar use of medical knowledge in both northern and southern populations. The relative importance of this plant species reflected the degree of agreement in the use of a medicinal plant. A medicinal plant with high frequency of citation, derived from informants of different ethnicities, is more likely to have clinical effect.

The rhizome extract of *Zingiber purpureum* had the second highest activity. *Zingiber purpureum,* or “phlai”, was previously known under the names *Zingiber montanum* (J.Koenig) Link ex A. Dietr and *Zingiber cassumunar* Roxb. (cassumunar ginger). The rhizome extracts had high ACE inhibitor activity. The ethanolic rhizome extracts at concentrations of 0.4, 2.0, and 10.0 mg/mL tended to have moderate inhibitory activity of 34, 39, and 40%, respectively. The aqueous rhizome extract at a concentration of 10 mg/kg BW had anti-hypertensive activity by decreasing the mean arterial blood pressure, which was up to 3.54 times more active than the standard drug. Bioactive compounds with anti-hypertensive effects had previously been reported, e.g., alkaloid and flavonoid from rhizome [26]. In northern Thailand in Chiang Mai province, *Z. purpureum* was prominently used for treating HT in several villages inhabited by four different ethnic groups (Hmong, Karen, Tai Yuan, and Tai Yai) [16]. Also in other regions, folk healers used *Z. purpureum* rhizome for treating hypertension such as in Songkhla province [25].

*Rotheca serrata* and *Clerodendrum chinense* had lower activity in our study compared to *Clerodendrum chinense* and *Zingiber purpureum*. *Rotheca serrata*, or “tri-chawa”, was previously known as *Clerodendrum serratum* (Linn.) Moon. In northern Thailand in Chiang Mai province, *R. serrata* was used for treating HT in many villages inhabited by three different ethnic groups (Karen, Lisu, and Tai Yai) [16]. Our data showed that *R. serrata* leaf extracts had a very poor inhibitory effect on ACE activity with the percentage of ACE inhibitory activity between 1.9–9.2% (Figure 1). However, our findings were consistent with previous reports about its ACE inhibitory activity (Table 1). The root extracts presented ACE inhibitory effects [27,28,29]. Moreover, the leaf extracts had vasorelaxant activity [27]. There was a previous report that phenolic compounds from this plant had anti-hypertensive effects [30].

*Clerodendrum chinense,* or “Ping-hom”, was previously known as *Clerodendrum chinense* (Osbeck) Mabb. var. *simplex* (Moldenke) S. L. Chen. In northern Thailand in Chiang Mai province, *C. chinense* was prominently used for treating HT in many villages inhabited by four different ethnic groups (Hmong, Karen, Tai Yuan, and Tai Yai) [16]. *Clerodendrum* *chinense* leaf extracts had a very poor inhibitory effect on ACE activity between 2.7–34% (Figure 1). However, the negative result of the ACE inhibition screening does not always mean that this plant does not work as anti-hypertensive drug as its compounds may influence other hypotensive mechanism. Nevertheless, the results related to the aqueous extract were consistent with previous reports about its anti-hypertensive activity (Table 1). The aqueous extract, which was prepared from the combination of leaves, stem and roots of *C. chinense,* did have anti-hypertensive activity [31]. The bioactive compounds with anti-hypertensive effects that were found in the extract prepared from the combination of leaves, stems, and roots included flavonoids, tannins, saponins, terpenoids, phlobatanins, cardiac glycosides, and phytosterols [31].

Notably, in the present study the preliminary safety profile of the extract was evaluated using HEK293T cells as a model for acute cytotoxicity assessment. HEK293T cells were selected because the kidney is one of the major organs involved in detoxification and is particularly susceptible to toxic insult following exposure to bioactive compounds. Cell viability was determined by measuring the redox capacity of metabolically active cells and provides a sensitive indicator of cellular viability. However, toxicity evaluation using a single cell type may not fully represent the overall safety profile of the extract. Additional assessment using other physiologically relevant cell types, particularly hepatocytes and vascular cells, together with complementary toxicity assays, i.e., apoptosis assay and proliferation assay, would provide a more comprehensive understanding of potential toxicological effects and improve the translational relevance of the findings. Therefore, further investigation is warranted to better characterize the safety profile of the extract for future therapeutic application. Furthermore, the additional studies should be undertaken to confirm several important points of the four selected plants, including ACE domain target selectivity (relevant for blood pressure reduction), detailed evaluation of plasma ACE and tissue specific ACE (e.g., endothelial or renal), characterization of inhibitory mechanisms (e.g., competitive, noncompetitive, or via chelation) of ACE inhibitory activity and the multi target mechanisms tests with other renin–angiotensin–aldosterone system (RAAS) to provide a broader understanding and discussion of the study.

**Table 2:** Plant screened for angiotensin I-converting enzyme inhibitory activity.

Species	*Blumea balsamifera*	*Clerodendrum chinense*	*Rotheca serrata*	*Zingiber purpureum*
**Family**	Asteraceae	Lamiaceae	Lamiaceae	Zingiberaceae
**Voucher number**	202223	202205	202220	202322
**Phenological stage**	Vegetative	Reproductive	Reproductive	Maturation
**Ethnic groups**	Hmong, Karen, Lisu, Tai Yai, and Tai Yuan	Hmong, Karen, Tai Yuan, and Tai Yai	Karen, Lisu, and Tai Yai	Hmong, Karen, Tai Yai, and Tai Yuan
**Part used**	Leaves	Leaves	Leaves	Rhizome
**Phytochemical characterization**	**ACE inhibition assay (leaves):** Flavonoids [6,24], Terpenoids [6], Luteolin [24], Quercetin [24]	**Antioxidant capacity assay (leaves):** Hydrolysable tannins, flavonoids, terpenoids, saponins [30] **Anti-hypertensive assay (mixed of leaves, stem and root):** Flavonoids, tannins, saponins, terpenoids, phlobatanins, cardiac glycosides, and phytosterols [25]	**Anti-hypertensive assay (leaves):** Phenolic compounds [26], polyphenolics (hydrolysable tannins and flavonoids), terpenoids, saponins [30]	**Anti-hypertensive assay (rhizome):** Alkaloid and flavonoid [27]
**Anti-hypertensive activity reported**	Tea preparation of leaves showed inhibitory activity on rabbit lung ACE [6], Ethanolic extract of leaves showed potential as anti-hypertensive agents in animal test models induced by epinephrine [24]	Aqueous extract of mixed leaves, stem and root showed the decrease in systolic, diastolic blood pressure [25]	Inhibiting angiotensin-converting enzyme [28,29,30], Vasorelaxant activity of leaf extract [30]	Aqueous rhizome extracts decrease of arterial blood pressure [27]

## 4. Materials and Methods

### 4.1. Preparation of Plant Materials

**Figure 6:**
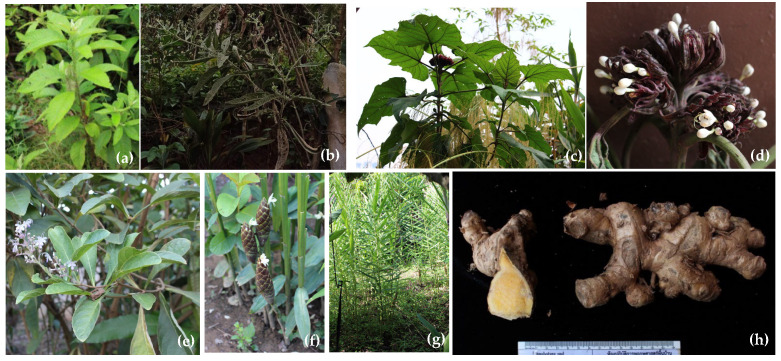
Four plant species screened for angiotensin I-converting enzyme inhibitory activity screening: (**a**,**b**) *Blumea balsamifera*, (**c**,**d**) *Clerodendrum chinense*, (**e**) *Rotheca serrata*, and (**f**–**h**) *Zingiber purpureum.*

Four plants (Table 2, Figure 6) were collected during fieldwork in Chiang Mai Province from November 2022 to January 2024. The criteria for selection of our study sites for fieldwork reflected a cultural bias based on previous research [14,16], such as (1) location of the villages must be far from each other to prevent the impact of horizontal knowledge exchange, (2) at least 90% of villagers were of the same ethnicity, as informed by the local government and the head of the village, (3) the villages should have been established at least 50 years ago so their home-gardens were well established and in a mature state, and (4) the villages were not located near urban centers and they were separated from other ethnic groups and from each other to minimize the effects of urbanization and cultural exchange. However, criteria numbers (2) and (3) were to ensure that the villages were representative of ethnicity. Vouchers of the plants were identified by the authors using taxonomic literature, including Flora of Thailand, Flora of China, Flora of Java, and Thai Forest Bulletin. Specimens were deposited at the Queen Sirikit Botanic Garden Herbarium (QBG), Chiang Mai, Thailand. The fresh plant specimens were thoroughly cleaned with tap water to remove dirt and impurities before they were used to screen for their anti-hypertensive properties. The water was drained from the plant materials by air drying in the shade at room temperature for three hours. The samples were pounded into powder with an electronic grinder.

### 4.2. Preparation of Plant Extracts for Screening of ACE Inhibitory Activity

The plant material (250 g) was macerated in 95% ethanol (500 mL) on a shaker for 24 h. Then, the ethanolic crude extract was filtrated through a filter paper using the Buchner funnel. The plant materials were macerated and filtrated three times. The ethanolic solvent was removed from the crude extract using a rotary evaporator with the setting at 100 mbar pressure and 50 °C temperature. Next, the ethanolic crude extract was transferred in amber glass bottles and kept at −20 °C temperature before freeze-drying. The ethanolic crude extract was placed in the freeze-dryer with setting 0.0 mbar pressure and −50 °C temperature for 3–7 days. After that, the crude extract was kept in glass bottles and sealed with the parafilm wax and aluminum foil to avoid the moisture and light consecutively. The bottles of crude extract were kept at −20 °C temperature.

### 4.3. Preparation of the Stock Solution

The 200 mg crude extract was dissolved in 1 mL dimethyl sulfoxide in a microcentrifuge tube. The microcentrifuge tube was sealed with parafilm wax before mixing. The microcentrifuge tube of crude extract solution was mixed with the sonication on the ultrasonic bath with the setting of 50 °C temperature for 30 min per cycle. Each process of sonication was repeated three times. The stock solution was stored at −20 °C temperature.

### 4.4. ACE1 Inhibitor Screening Kit Analysis

The stock of extract solution was dissolved in the phosphate buffered saline (PBS) solution and diluted into concentrations of 0.08, 0.4, 2.0, and 10.0 mg/mL, consecutively. Then, the diluted extract solutions were tested with ACE1 inhibitor screening kit (Abcam company, Milpitas, California, USA) by following the manufacturer’s instructions. The preparation method followed the datasheet information ( Available online: http://www.abcam.com/ab283407 [accessed on 12 June 2026]). The ACE1 enzyme was derived from recombinant human protein. The samples included the test extracts (S), 0.2 mg/mL (1 μM) of captopril as positive inhibitor control (IC), 2 μL of ACE1 enzyme as enzyme control (EC), background control containing no enzyme (BC), and 99% PBS + 1% DMSO as solvent control (SC) in a 96-well UV transparent plate. The diluted ACEI enzyme was added into the wells of (S), (IC), (EC), and (SC) and further incubated at the 37 °C temperature for 15–20 min with light protecting to allow the interaction of ACEI enzyme and inhibitors. After the ACEI substrate was added to each well, the absorbance was measured immediately at the OD 345 nm in the kinetic mode of microplate reader for 60 min at 37 °C temperature. Notably, to avoid interference caused by plant pigments, the absorbance of the extract alone was independently measured and used as background correction. The samples were divided into three plates and then the absorbance values of each plate were measured independently. For the statistical analysis, the absorbance values were obtained from these three biological duplicates (*n* = 3). We used two time points (t1 and t2) in the linear range of the plot and the corresponding absorbance values (OD1 and OD2) were used to calculate the slope by dividing the ∆OD = (OD1 − OD2). The slope was calculated to find the % relative inhibition by comparing it with the enzyme control using the following equation:

Percentage (%) of relative inhibition = [(Slope of enzyme control −  Slope of sample)/Slope of enzyme control] × 100

The result was normalized by % relative inhibition of background control to confirm the presence of substrate under the experimental condition before the analysis. Finally, the OD values were analyzed with the unpaired t-test by comparing the % relative inhibition of sample extract and enzyme control which was set at the 100% inhibition. The significant difference was considered at *p*-value < 0.05.

The IC_50_ value was defined as the concentration of inhibitor required to inhibit 50% of the ACE activity under the assay conditions and was determined by regression analysis of the ACE inhibition (%) versus the log of the inhibitor concentration.

### 4.5. Cytotoxicity Analysis

HEK293T cells (American Type Culture Collection, Manassas, VA, USA) were seeded into a 96-well plate (15,000 cells/well) a day before the experiment. Various concentrations of the *Clerodendrum chinense*, *Rotheca serrata*, *Zingiber purpureum*, and *Blumea balsamifera* extracts were prepared (0.64–2000 μg/mL) in media and added to the cells. The cell viability was determined after 24 and 48 h of incubation by using PrestoBlue™ cell viability reagent (Invitrogen, Waltham, MA, USA). Absorbance was recorded at 570 nm and 595 nm using a microplate reader (Hangzhou Allsheng, Hangzhou, China) and used to calculate the % cell viability relative to that of non-treatment control using the following equation:

Percentage (%) of cell viability = [(OD570 − OD595) Test/(OD570 − OD595) Control] × 100

## 5. Conclusions

The ethanolic extracts of the four prominent species used for treating hypertension in northern Thailand with the high degree of agreement value among the ethnic communities were tested for their inhibitory activity against an angiotensin-converting enzyme (ACE) using bio-screening of a colorimetric assay technique. The ethanolic extracts of *Blumea balsamifera* leaves and *Zingiber purpureum* rhizomes showed considerable ACE inhibitor activity. This suggests that medicinal plants traditionally used by multiple cultures in hypertension treatment surely have a pharmacological effect, and the data provided here may lead to the discovery of new anti-hypertensive drugs. However, they should be further studied to confirm local use. The negative result of the ACE inhibition screening does not always mean that this plant does not work as anti-hypertensive drug as its compounds may influence other hypotensive mechanisms. In vivo studies and clinical investigations to validate the anti-hypertensive potential of plant species should be carried out.

## Data Availability

The original contributions presented in this study are included in the article/Supplementary Material. Further inquiries can be directed to the corresponding author.

## Supplementary Materials

The following supporting information can be downloaded at: https://www.mdpi.com/article/10.3390/plants15132068/s1, Table S1: The detail of medicinal plant species of Thai medicine recipe Kam Lung Rad Cha Si reported in two formulations as decoction method and powdering method data.

## References

[1] World Health Organization (2019). Hypertension Care in Thailand: Best Practices and Challenges 2019.

[2] Thai Hypertension Society (2019). Thai Guidelines on The Treatment of Hypertension 2019.

[3] Wong M.K.S., Takei Y., Ando H. (2015). Subchapter 29A—Renin. Handbook of Hormones: Comparative Endocrinology for Basic and Clinical Research.

[4] Ahnfelt R.I., Krogsgaard L.P., Bundgaard H. (1991). Subchapter-Enzyme inhibitor as drugs. A Textbook of Drug Design and Development.

[5] Oparil S., Zaman M.A., Calhoun D.A. (2003). Pathogenesis of hypertension. Ann. Intern Med..

[6] See G.L.L., Arce F.V., Deliman Y.C. (2016). ACE (angiotensin converting enzyme) inhibition activity of oven–dried and air–dried Sambong *Blumea balsamifera* L. (dc.) tea. Int. J. Pharmacogn. Phytochem. Res..

[7] Buranakitjaroen P., Sriussadaporn S., Phoojaroenchanachai M., Sangrasert P., Saravich S. (2003). Angiotensin converting enzyme inhibitor induced cough: Experience in Siriraj Hospital. J. Med. Assoc. Thai..

[8] Saiz L.C., Gorricho J., Garjon J., Celaya M.C., Erviti J., Leache L. (2022). Blood pressure targets for the treatment of people with hypertension and cardiovascular disease. Cochrane Database Syst. Rev..

[9] Feng Y., Zhou J., Zhong M., Ma D., Mao J., Liu F., Jiang C., Wu X., Jiang L. (2025). Plant-Derived Natural Products Ameliorating Hypertension via Signaling Pathways: A Review. Am. J. Chin. Med..

[10] Tabassum N., Ahmad F. (2011). Role of natural herbs in the treatment of hypertension. Pharmacogn. Rev..

[11] Ahmad I., Yanuar A., Mulia K., Mun’im A. (2017). Review of Angiotensin-converting Enzyme Inhibitory Assay: Rapid Method in Drug Discovery of Herbal Plants. Pharmacogn. Rev..

[12] Anuar T.A., Ismail A. (2020). Southeast Asian Medicinal Plants with Angiotensin Converting Enzyme (ACE) Inhibition Properties. Pharmacogn. J..

[13] Li J., Hu C., Zhu J.H., Li R.L. (2026). Multifaceted mechanisms of plant metabolites in pulmonary arterial hypertension: A critical review beyond vasodilation. Front. Pharmacol..

[14] Srithi K., Trisonthi C., Inta A., Balslev H. (2019). Cross-cultural Comparison of Medicinal Plants Used to Treat Infections in Northern Thailand. J. Econ. Bot..

[15] Phumthum M., Balslev H. (2019). Use of Medicinal Plants Among Thai Ethnic Groups: A Comparison. J. Econ. Bot..

[16] Sumridpiem P., Balslev H., Tiensawat P., Neamsuvan O., Inta A. (2025). Plants Used for Treating Hypertension Among Ethnic Groups in Northern Thailand. Plants.

[17] Wiwatkunupakarn N., Aramrat C., Sanguanwai P., Choksomngam Y., Gilder M.E., Jiraporncharoen W., McGready R., Angkurawaranon C. (2024). The use of herbal medicine for hypertension in rural and urban Thailand: A cross sectional study. J. Herb. Med..

[18] Chaiyawatthanananthn P., Houngiam K., Prajuabjinda O., Itharat A. (2022). Angiotensin-Converting Enzyme Inhibitory and Antioxidant Activities of Kam-Lung-Rad-Cha-Si, A Thai Herbal Formulation, Extracts. AMJAM.

[19] On-Nom N., Thangsiri S., Inthachat W., Temviriyanukul P., Trisonthi P., Chupeerach C., Siriwan D., Suttisansanee U. (2023). Phenolic profiles and in vitro biochemical properties of Thai herb ingredients for chronic diseases prevention. Sci. Rep..

[20] Madaka F., Pathompak P., Sakunpak A., Monton C., Charoonratana T. (2017). Angiotensin I-converting enzyme inhibitor activity of some medicinal plants listed in traditional Thai medicine. Interprofessional J. Health Sci..

[21] Simaratanamongkol A., Umehara K., Noguchi H., Panichayupakaranant P. (2014). Identification of a new angiotensin-converting enzyme (ACE) inhibitor from Thai edible plants. Food Chem..

[22] Office of International Cooperation (2016). Department for Development of Thai Traditional and Alternative Medicine, Thai Traditional Medicine at a Glance.

[23] Department of Thai Traditional and Alternative Medicine in Collaboration with the Food and Drug Administration The Reference Database List of Thai Traditional Medicine Formulary for Herbal Products Registration, Bangkok, Thailand, 2023. https://dmsic.moph.go.th/index/detail/9480.

[24] Sjahriza A., Shellia F., Iswantini D. (2023). Active Compounds of Sembung Leaves (Blumea balsamifera DC) in Silico Screening as Antihypertensives. J. Kim. Mulawarman.

[25] Odimegwu J.I., Okanlawon T.F., Olayunji O.E., Ismail I. (2024). Diuretic and Anti-Hypertensive Activity of Clerodendrum Chinense (OSBECK) MABB. Aqueous Extract in 8% Salt Diet-Induced Hypertensive Rats. Adv. J. Uro. Nephro..

[26] Singh M.K., Khare G., Iyer S.K., Sharwan G., Tripathi D.K. (2012). Clerodendrum serratum: A clinical approach. J. App. Pharm. Sci..

[27] Manosroi A., Lohcharoenkal W., Khonsung P., Manosroi W., Manosroi J. (2013). Potent antihypertensive activity of Thai-Lanna medicinal plants and recipes from MANOSROI III database. J. Pharm. Bio..

[28] Nyman U., Joshi P., Madsen L.B., Pedersen T.B., Pinstrup M., Rajasekharan S. (1998). Ethnomedical information and in vitro screening for angiotensin-converting enzyme inhibition of plants utilized as traditional medicines in Gujarat, Rajasthan and Kerala (India). J. Ethnopharmacol..

[29] Narayanan N., Thirugnanasambantham P., Viswanathan S., Vijayasekaran V., Sukumar E. (1999). Antinociceptive, antiinflammatory and antipyretic effects of ethanol extract of Clerodendron serratum roots in experimental animals. J. Ethnopharmacol..

[30] Mohamed A.J., Mohamed E.A.H., Abdalrahim F.A.A., Ameer O.Z., Ismail Z., Ismail N., Shah A.M., Abdulmajid M.Z.A., Yam M.F. (2012). Antioxidant, antiangiogenic and vasorelaxant activities of methanolic extract of Clerodendrum serratum (Spreng.) leaves. J. Med. Plants Res..

[31] Neamsuvan O., Komonhiran P., Boonming K. (2018). Medicinal plants used for hypertension treatment by folk healers in Songkhla province, Thailand. J. Ethnopharmacol..

