# Supplementary material for: Angiotensin I-Converting Enzyme Inhibitor Activity of Some Plants Used in Thai Indigenous Medicine
Source: Plants (Basel). 2026 Jul 3;15(13):2068. doi: 10.3390/plants15132068 (PMC13364157; doi:10.3390/plants15132068)
Supplement: Supplementary file 1 [file plants-15-02068-s001.zip › plants-4356977-supplementary-updated.pdf]

Supplementary Material

Table S1. The detail of medicinal plant species of Thai medicine recipe Kam Lung Rad Cha Si reported in two formulations as decoction method and powdering method.

Thai traditional name	Species name as in original reference	Updated species name*	Preparation method	
			Decoction	Powdering
Cha lud	1. *Alyxia reinwardtii* Blume	*Alyxia reinwardtii* Blume	Bark of stems	×
Krit sa na	2. *Aquilaria crassna* Pierre ex Lecomte[1]	*Aquilaria crassna* Pierre ex Lecomte	Heartwood	Heartwood
Kot chu la lampha	3. *Artemisia annua* L.	*Artemisia annua* L.	Arial parts	×
Kamlang wua tha loeng	4. *Anaxagorea luzonensis* A. Gray	*Anaxagorea luzonensis* A. Gray	×	Stems
Kot so	5. *Angelica dahurica* (Hoffm.) Benth. & Hook. f. ex Franch. & Sav.	*Angelica dahurica* (Hoffm.) Benth. & Hook.f. ex Franch. & Sav.	Roots	×
Kot chiang	6. *Angelica sinensis* (Oliv.) Diels	*Angelica sinensis* (Oliv.) Diels	Roots & Rhizomes	Roots & Rhizomes
Thian ta tak ka taen	7. *Anethum graveolens* L.	*Anethum graveolens* L.	Fruits	×
Kot kha mao	8. *Atractylodes lancea* (Thunb.) DC.	*Atractylodes lancea* (Thunb.) DC.	Rhizomes	×
Thao wan priang	9. *Brachypterum scandens* (Roxb.) Wight & Arn. ex Miq.	*Brachypterum scandens* (Roxb.) Wight & Arn. ex Miq.	×	Stems
Kham thai	10. *Bixa orellana* L.	*Bixa orellana* L.	Flowers	Flowers
Fang sen	11. *Caesalpinia sappan* L.	*Biancaea sappan* (L.) Tod.*	Heartwood	Heartwood
Kra dang nga	12. *Cananga odorata* (Lam.) Hook. f. et Thomson	*Cananga odorata* (Lam.) Hook.f. & Thomson	Flowers	Flowers
Sa mun waeng	13. *Cinnamomum bejolghota* (Buch.-Ham.) Sweet	*Cinnamomum bejolghota* (Buch.-Ham.) Sweet	×	Bark of stem
Op choei thet	14. *Cinnamomum verum* J.Presl	*Cinnamomum verum* J.Presl	Bark of stems	×
Op choei	15. *Cinnamomum iners* (Reinw. ex Nees & T.Nees) Blume[2]	*Cinnamomum iners* (Reinw. ex Nees & T.Nees) Blume	×	Bark of stem
Thian khao	16. *Cuminum cyminum* L.	*Cuminum cyminum* L.	Fruits	Fruits
Kra lam phak	17. *Excoecaria agallocha* L. [3]	*Excoecaria agallocha* L.	Heartwood	Heartwood
Thian khao plueak	18. *Foeniculum vulgare* Mill.	*Foeniculum vulgare* Mill.	Fruits	Fruits
Cha em thet	19.*Glycyrrhiza glabra* L.	*Glycyrrhiza glabra* L.	Roots	×
Kot so thet	20. *Iris x germanica* L. [4]	*Iris x germanica* L.	×	Roots
Ma li	21. *Jasminum sambac* (L.) Aiton	*Jasminum sambac* (L.) Aiton	Flowers	×
Thian daeng	22. *Lepidium sativum* L.	*Lepidium sativum* L.	Seeds	Seeds
Kot hua bua	23. *Ligusticum sinense* Oliv.	*Conioselinum anthriscoides* (H.Boissieu) Pimenov & Kljuykov*	Rhizomes	×
Kot hua bua	24. *Ligusticum sinense* Oliv. cv. Chuanxiong	*Conioselinum anthriscoides* (H.Boissieu) Pimenov & Kljuykov*	×	Rhizomes
Cham pa	25. *Magnolia champaca* (L.) Baill. ex Pierre	*Magnolia champaca* (L.) Baill. ex Pierre	Flowers	Flowers

Thai traditional name	Species name as in original reference	Updated species name*	Preparation method	
			Decoction	Powdering
Sa ra phi	26. *Mammea siamensis* T. Anderson	*Mammea siamensis* (Miq.) T.Anderson*	Flowers	×
Bun nak	27. *Mesua ferrea* L.	*Mesua ferrea* L.	Flowers	Flowers
Khon dok	28. *Mimusops elengi* L.[5]	*Mimusops elengi* L.	Heartwood	Heartwood
Phi kun	29. *Mimusops elengi* L.	*Mimusops elengi* L.	Flowers	×
Luk chan/ Dok chan	30. *Myristica fragrans* Houtt.	*Myristica fragrans* Houtt.	Seeds & Arils	Seeds & Arils
Kot kan phrao	31. *Neopicrorhiza scrophulariiflora* (Pennell) Hong	*Neopicrorhiza scrophulariiflora* (Pennell) D.Y.Hong*	×	Roots & Rhizomes
Bua luang/ Bua sat ta bong kot	32. *Nelumbo nucifera* Gaertn.	*Nelumbo nucifera* Gaertn.	Stamens	Stamens & Flowers
Thian dam	33. *Nigella sativa* L.	*Nigella sativa* L.	Seeds	Seeds
Bua khao	34. *Nymphaea ampla* (Salisb.) DC.	*Nymphaea ampla* (Salisb.) DC.	×	Flowers
Bua daeng	35. *Nymphaea lotus* L.	*Nymphaea lotus* L.	×	Flowers
Bua khom	36. *Nymphaea pubescens* Wild.	*Nymphaea pubescens* Willd.*	×	Flowers
Bua phuean	37. *Nymphaea nouchali* Burm.f.	*Nymphaea nouchali* Burm.f.	×	Flowers
Chet ta mun phloeng	38. *Plumbago indica* L.	*Plumbago indica* L.	Roots	×
Phrik thai	39. *Piper nigrum* L.	*Piper nigrum* L.	×	Fruits
Di pli	40. *Piper retrofractum* Vahl	*Piper retrofractum* Vahl	Fruits	Fruits
Cha phlu	41. *Piper sarmentosum* Roxb.	*Piper sarmentosum* Roxb.	Roots	×
Sa khan	42. *Piper wallichii* (Miq.) Hand.-Mazz.	*Piper wallichii* (Miq.) Hand.-Mazz.	Stems	×
Chan daeng/ Lak chan	43. *Pterocarpus santalinus* L. f.[6]	*Pterocarpus santalinus* L. f.	Heartwood	×
Chan khao	44. *Santalum album* L.	*Santalum album* L.	Heartwood	×
Kan phlu	45. *Syzygium aromaticum* (L.) Merr. & L.M.Perry	*Syzygium aromaticum* (L.) Merr. & L.M.Perry	Flowers	Flowers
Kot phung pla	46. *Terminalia chebula* Retz.	*Terminalia chebula* Retz.	×	Galls
Ya nang	47. *Tiliacora triandra* (Colebr.) Diels.	*Tiliacora triandra* (Colebr.) Diels.	×	Roots
Thian yao wa pha nee	48. *Trachyspermum ammi* (L.) Sprague[7]	*Trachyspermum ammi* (L.) Sprague	×	Roots
Muak khao	49. *Urceola laevigata* (Juss.) D. J. Middleton & Livsh.	*Urceola laevigata* (Juss.) D. J. Middleton & Livsh.	×	Stems
Muak daeng	50. *Urceola rosea* (Hook. & Arn.) D. J. Middleton	*Urceola rosea* (Hook. & Arn.) D. J. Middleton	×	Stems
Kra wan	51. *Wurfbainia testacea* (Ridl.) Skornick. & A.D.Poulsen[8]	*Wurfbainia testacea* (Ridl.) Skornick. & A.D.Poulsen	Fruits	×

Thai traditional name	Species name as in original reference	Updated species name*	Preparation method	
			Decoction	Powdering
Khing/ Khing haeng	52. *Zingiber officinale* Roscoe	*Zingiber officinale* Roscoe	Rhizomes	Rhizomes

Species name of the Thai medicine recipe Kam Lung Rad Cha Si reported from "The reference database list of Thai Traditional Medicine Formulary for herbal products registration" [23]. The medicinal plant species were interchangeable based on shared therapeutic properties in Thai traditional practice: [1]*Aquilaria malaccensis* Lam., [2]*Cinnamomum verum* J. Presl, [3]*Euphorbia antiquorum* L., [4]*Iris pallida* Lam., [5]*Lagerstroemia floribunda* Jack, [6]*Dracaena cochinchinensis* (Lour.) S. C. Chen, [7]*Petroselinum crispum* (Mill.) Fuss, [8]*Wurfbainia vera* (Blackw.) Skornick. & A. D. Poulsen. Scientific name updated to follow *World Flora Online* (Available online: https://www.worldfloraonline.org/ [accessed on 12 June 2026]).
